# The add-on effect of Shufeng Jiedu capsule for treating COVID-19: A systematic review and meta-analysis

**DOI:** 10.3389/fmed.2022.1020286

**Published:** 2022-10-13

**Authors:** Xiaodi Sheng, Chao Chen, Guowang Jiang, Zhaochen Ji, Zehui Guo, Haiyin Hu, Hui Wang, Jingbo Zhai, Dong Zhang, Junhua Zhang, Liping Guo

**Affiliations:** ^1^Evidence-Based Medicine Center, Tianjin University of Traditional Chinese Medicine, Tianjin, China; ^2^First Teaching Hospital of Tianjin University of Traditional Chinese Medicine, Tianjin, China; ^3^Tianjin University of Traditional Chinese Medicine, Tianjin, China; ^4^Tianjin Academy of Traditional Chinese Medicine Affiliated Hospital, Tianjin, China

**Keywords:** COVID-19, Shufeng Jiedu capsule, traditional Chinese medicine, systematic review, meta-analysis

## Abstract

**Introduction:**

Shufeng Jiedu capsule (SFJD) is a commonly used Chinese patent medicine in China. Some studies have reported that SFJD has therapeutic effects in patients diagnosed with COVID-19. This systematic review aimed to critically evaluate the efficacy and safety of SFJD combined with western medicine (WM) for treating COVID-19.

**Methods:**

A literature search by using WHO COVID-19 database, PubMed, Embase, Cochrane Library, the Web of Science, CKNI, Wanfang, VIP, SinoMed, and clinical trial registries was conducted, up to 1 August 2022. Randomized controlled trials (RCTs), non-RCTs, cohort studies and case series of SFJD combined with WM for COVID-19 were included. Literature screening, data extraction, and quality assessment were performed independently by two reviewers in line with the same criteria. We used the Grading of Recommendations, Assessment, Development, and Evaluations (GRADE) to assess the certainty of evidence. Meta-analyses were performed with Revman 5.3 if possible. The descriptive analysis was conducted when the studies could not be meta-analyzed.

**Results:**

Totally 10 studies with 1,083 patients were included. Their methodological quality were moderate. The results demonstrated that compared to WM group, SFJD + WM group remarkably increased the nucleic acid negative conversion rate (*RR* = 1.40, 95%CI: 1.07–1.84), total effective rate (*RR* = 1.18, 95%CI: 1.07–1.31), cure rate (*RR* = 4.06, 95%CI: 2.19–7.53), and the chest CT improvement rate (*RR* = 1.19, 95%CI: 1.08–1.31), shorten nucleic acid negative conversion time (*MD* = −0.70, 95%CI: −1.14 to −0.26), reduced the clinical symptom disappearance time (fever, diarrhea, cough, fatigue, pharyngalgia, nasal congestion, and rhinorrhea), as well as improved the levels of laboratory outcomes (CRP, IL-6, Lym, and Neu). Additionally, the incidence of adverse reactions did not exhibit any statistically significant difference between SFJD + WM group and WM group.

**Conclusion:**

SFJD combined with WM seems more effective than WM alone for the treatment of COVID-19. However, more well-designed RCTs still are warranted.

**Systematic review registration:**

[https://www.crd.york.ac.uk/PROSPERO/], identifier [CRD42022306307].

## Introduction

Coronavirus disease 2019 (COVID-19) is an acute respiratory infectious disease caused by severe acute respiratory syndrome coronavirus-2 (SARS-CoV-2) (1, 2), which features a high infection rate. Fever, cough, dyspnea, fatigue, and other symptoms of pneumonia are the major symptoms among COVID-19 patients (3). On 11 March 2020, COVID-19 is defined by the World Health Organization (WHO) as the worldwide pandemic. By the time of 14 April 2022, totally 500.19 million confirmed COVID-19 cases and 6.19 million death cases were reported globally (4). Undoubtedly, COVID-19 greatly threatens human health worldwide. Therefore, it is urgent to find new drugs or new treatments for controlling the replication and spread of SARS-CoV-2.

Traditional Chinese medicine (TCM) has been applied in China for thousands of years (5), with the characteristics of holistic concept and syndrome differentiation (6). It is featured with specific merits in preventing, treating and taking health care of different disorders (7). The fight against COVID-19 has verified the effectiveness of TCM on treating infectious disorders (8, 9). In line with results reported by the National Health Commission of the People’s Republic of China, 91.5% of the Chinese confirmed COVID-19 cases received TCM combined with Western medicine (WM) treatment (10). In addition, the report of the “WHO Expert Meeting on Evaluation of Traditional Chinese Medicine in the Treatment of COVID-19” pointed out that TCM can effectively treat COVID-19, decrease the conversion time of nucleic acid, and reduce the severe/critical case rate, while improving mild and ordinary patient prognosis (11).

As one of the frequently applied Chinese patent medicines, Shufeng Jiedu Capsule (SFJD) has been used to treat influenza in China. Studies have demonstrated that SFJD exerts antiviral, antibacterial, and immune-enhancing effects (12). Researches suggest that enhancing the host immune response to RNA virus infection is critical for treating COVID-19 cases (13). SFJD can be considered as an option for treating COVID-19 (14).

To date, some studies have reported that SFJD exerts therapeutic effects in patients diagnosed with COVID-19. Therefore, we conducted this systematic review and meta-analysis to critically assess whether SDJF + WM improved effective outcomes, clinical symptoms outcomes and laboratory outcome, as well as generated less adverse medication effects compared to WM in the treatment of COVID-19 patients.

## Methods

This study was conducted in line with statement of Preferred Reporting Items for Systematic Reviews and Meta-Analyses (PRISMA) (15), and has been prospectively registered on the International Prospective Register of Systematic Reviews (PROSPERO: CRD42022306307).

### Eligibility criteria

#### Inclusion criteria

1. Patients: Patients who were diagnosed with COVID-19 in accordance with the recognized diagnostic criteria including the “Diagnosis and Treatment Protocol for Novel Coronavirus Pneumonia” (14) were included in this study, and their country, age, gender, and course of disease were not limited.
2. Intervention: Patients in the experiment group of the trials, the exposure group of the cohort studies or the case series were treated with SFJD alone or SFJD in combination with WM.
3. Comparison: Patients in the control group of trials or the non-exposed group of cohort studies were given WM.
4. Outcome: Based on the COVID-19 core outcome sets (COS) (16), efficacy outcomes (including nucleic acid negative conversion rate and time, total effective rate, cure rate, turning to severe/critical illness rate, chest computerized tomography (CT) improvement rate, and length of hospital stay), clinical symptom outcomes (including cough, fatigue, fever, etc.), laboratory outcomes (including CRP, IL-6, LYM, WBC, etc.) and safety outcomes (nausea, sour regurgitation, allergic reaction, etc.) were considered for the analysis.
5. Study types: Randomized controlled trials (RCTs), non-RCTs, cohort studies, and case series were included.

#### Exclusion criteria

1. Studies in which patients taking other traditional Chinese medicine instead of SFJD in the experimental group were excluded.
2. Studies without complete data were excluded.
3. Duplicate publications were excluded.

### Search strategy

The following electronic databases were involved: the WHO COVID-19 database (17), PubMed, Embase, Cochrane Library, the Web of Science, the China National Knowledge Infrastructure (CKNI), the Wanfang Data Knowledge Service platform, the VIP information resource integration service platform, and SinoMed, until 1 August 2022. In terms of search terms, “Shufeng Jiedu,” “Shufengjiedu,” “COVID-19,” “2019-nCov,” “SARS-CoV-2,” “2019 novel coronavirus,” and “NCP” were contained. The details of search strategies are shown in Supplementary Table 1. Additionally, we also supplemented the search with clinical trial registries (ChiCTR, Clinicaltrials.gov, WHO ICTRP, and PROSPERO). Besides, references to the included literature were traced back, aiming to add the acquisition of relevant literature.

### Study selection and data extraction

This study used NoteExpress (3.2.0) software in managing records while removing duplicate ones. In the current work, two researchers (CC and XS) were responsible for the independent literature screening in line with our pre-set inclusion/exclusion criteria. Firstly, titles and abstracts of studies were read to exclude the apparently unrelated studies. Secondly, full-texts were carefully read to examine its eligibility. Any disagreement between them was solved by the opinion of a third researcher (DZ).

Two reviewers (HH and GJ) independently extracted and cross-checked data using predesigned standard data extraction forms. The information of the form included basic information, participants’ baseline characteristics, details of experiment and control groups, as well as outcomes (such as event number, overall participant number, mean ± standard deviation for continuous data, overall subjects for dichotomous data). Any disagreement was settled down by discussing or negotiating with a third party (HW). Moreover, we also contacted the corresponding authors to supplement the missing data.

### Risk of bias and methodological quality assessments

The risk of bias and methodological quality of the included studies was evaluated by two researchers (XS and ZJ) independently, and disagreements between them were settled down by discussing with a third researcher (JHZ). The risk of bias of RCTs was assessed by the Cochrane Risk of Bias (ROB) assessment tool (18), when each RCT was evaluated as “low,” “high,” or “unclear” bias risk according to seven areas below, including generation of random sequence, concealment of allocation, personnel and participant blinding, outcome evaluation blinding, insufficient outcome measure, selective report of outcomes, as well as other bias. The Newcastle-Ottawa Scale (NOS) (19) consists of eight items classified as 3 dimensions, including population selection, outcome evaluation and component comparability, was utilized to evaluate the methodological quality of non-RCTs and cohort studies. Additionally, the Institute of Health Economics (IHE) quality appraisal (QA) checklist (20) was utilized to assess the methodological quality of case series, involving the study objective, research design, objects of study, intervention/cointervention (s), outcome measures, statistical analyses, results and conclusions, conflict of interests, and financial support sources.

### Certainty of evidence assessment

Two reviewers (XS and CC) independently assessed the certainty evidence of meta-analysis outcomes using the Grading of Recommendations Assessment, Development and Evaluation (GRADE) system (21), which categorized overall quality of evidence as “high,” “moderate,” “low,” and “very low” certainty. Any disagreements were resolved by mutual consensus or by consulting a third reviewer (JBZ). The starting point for the certainty for RCTs was “high,” while it was “low” for observational studies. Five downgraded factors were considered in the evidence assessment, including the risk of bias, inconsistency, indirectness, imprecision, and publication bias. The three upgrading factors included larger effect, dose-response gradient, and effect of plausible confounding.

### Statistical analysis

In this study, the RevMan 5.3 software was employed for meta-analysis. Dichotomous data were analyzed by risk ratio (RR), and continuous data were denoted by mean difference (MD), whereas each type of effect size was determined by 95% confidence interval (CI). In line with Cochrane Handbook of Systematic Evaluation of Interventions (22), this study estimated those missing information. *I*^2^ statistic, *P*-value, and Tau^2^ were used to evaluate statistical heterogeneity (23). When *I^2^* ≤ 50% and *P* > 0.1 indicated low heterogeneity between those enrolled articles, the fixed-effect model would be selected for analysis. By contrast, high heterogeneity existed in the case of *I^2^* > 50% or *P* ≤ 0.1, and thus the random-effect model would be selected for meta-analysis. Moreover, we also conducted subgroup (e.g., disease severity) and sensitivity analyses to explore potential heterogeneity sources when sufficient data were available. In order to determine possible heterogeneity sources, R (version 4.0.5) was used to perform meta-regression on these outcomes (the number of included studies >3 and *I*^2^ >50%). We selected multiple covariants to verify potential heterogeneity, involving sample size, language, and male-female ratio. If more than 10 studies were included, a funnel plot was drawn to identify publication bias (24).

## Result

### Study screening

**FIGURE 1:**
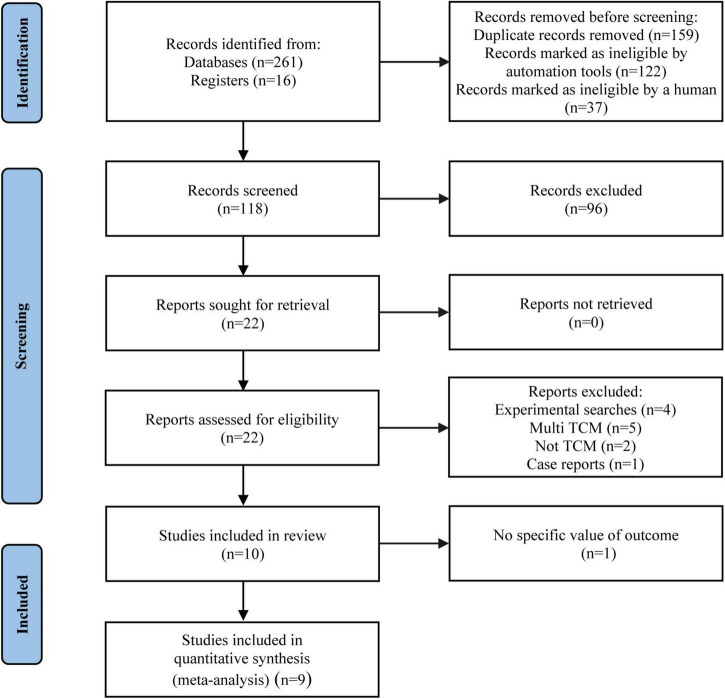
PRISMA flow diagram of study selection process.

During the initial search, 277 records were identified from nine databases and clinical trial registries. After 159 duplicates were removed, 118 studies were screened by the titles/abstracts. Afterward, we read the full-texts of 22 articles (Supplementary Table 2). Totally 10 articles were eligible for inclusion, while 9 articles (25–33) were enrolled for finally analysis, since one study (34) reported no specific value of the outcome, causing inability to merge data. The literature screening procedure is displayed in Figure 1.

### Study characteristics

Table 1 presents the general information of the involved studies. Totally 10 studies were included, and were published from 2020 to 2022 with 3 in English and 7 in Chinese. Specifically, two studies was RCTs (25, 33), one study was a non-RCT (26), five were retrospective cohort studies (28–32), and two were case series studies (27, 34). Additionally, a total of 1,083 patients were involved in the study, and the sample size of each study ranged from 11 to 234 cases, with an average sample size of 108 cases. COVID-19 patients’ disease staging is mainly mild and ordinary. A summary table of SFJD details for each study is provided in Supplementary Table 3. The details the diagnostic criteria of included study were listed in Supplementary Table 4.

**TABLE 1:** General characteristics of the included studies.

Study	Type of study	COVID-19 stage	Sample size (E/C)	Age (M ± SD) (E/C)	Treatment		Dose/Frequency (SFJD)	Durations
					Intervention	Control		
Yan et al. (25)	RCT	Ordinary	50/50	60.26 ± 7.32/59.48 ± 8.24	SFJD + interferon alpha + arbidol	Interferon alpha + arbidol	2.08 g/times, tid	14 days
Zhang et al. (33)	RCT	Mild	117/117	41.7 ± 9.9/41.2 ± 13.9	SFJD + conventional therapy	Conventional therapy	2.08 g/times, tid	7 days
Chen et al. (30)	Cohort study	Ordinary	34/34	65.06 ± 10.63/64.35 ± 10.34	SFJD + conventional therapy	Conventional therapy	2.08 g/times, tid	7 days
Qu et al. (28)	Cohort study	Mild and ordinary	40/40	39.65 ± 11.20/41.60 ± 10.50	SFJD + arbidol + conventional therapy	Arbidol + Conventional therapy	2.08 g/times, tid	10 days
Wu et al. (27)	Case series	Ordinary	44	43.04 ± 15.33	SFJD + conventional therapy		2.08 g/times, tid	3–7 days
Chen et al. (32)	Cohort study	Ordinary	100/100	60.2 ± 6.6/60.4 ± 6.6	SFJD + arbidol	Arbidol	2.08 g/times, tid	14 days
Xia et al. (31)	Cohort study	Mild and ordinary	43/33	46.95 ± 14.9/45.9 ± 13.3	SFJD + antiviral drugs (umifenovir /lopinavir/ritonavir tablets)	Antiviral drugs (umifenovir/ lopinavir/ritonavir tablets)	2.08 g/times, tid	Within 3 week
Guo et al. (34)	Case series	Ordinary and severe	11	40.5 (13.5–66)	SFJD + antiviral drugs (abidor/umifenovir/lopinavir tablets)		/	/
Qu et al. (29)	Cohort study	Mild and ordinary	40/30	40.65 ± 8.23/39.82 ± 6.40	SFJD + arbidol + conventional therapy	Arbidol + conventional therapy	2.08 g/times, tid	10 days
Xiao et al. (26)	Non-RCT	Mild	100/100	69.90 ± 8.70/62.20 ± 7.50	SFJD + arbidol	Arbidol	2.08 g/times, tid	14 days

E, Experiment group; C, Control group; M, Mean; SD, Standard Deviation; SFJD, Shufeng Jiedu capsule; tid, three times a day; RCT, Randomized controlled trial.

### Risk of bias and methodological quality assessment

Table 2 displays risk of bias of the enrolled RCTs (25, 33). Two studies describe the generation process of an appropriate random sequence, with “generation of random sequence” being considered to be of “low risk.” There were no missing data in either study and this work considered “incomplete outcome data” to be of “low risk.” Regarding blinding of participants and outcome reviewers, one study (33) was randomized open-label and rated as “high risk,” while the other (25) was not reported in the original text and was therefore “unclear risk.” Additionally, allocation concealment method, selective report of outcomes, and other biases were not reported in two articles, which were thus rated as “unclear risk.”

**TABLE 2:** The risk of bias of included randomized controlled trials.

Study	Random sequence generation (selection bias)	Allocation concealment (selection bias)	Blinding of participants and personnel (performance bias)	Blinding of outcome assessment (detection bias)	Incomplete outcome data (attrition bias)	Selective reporting (reporting bias)	Other bias
Yan et al. (25)	L	U	U	U	L	U	U
Zhang et al. (33)	L	U	H	H	L	U	U

L, Low risk; U, Unclear risk; H, High risk.

The NOS scale was adopted for assessing the methodological quality of the cohort studies and the non-RCTs. The highest rating is 9 stars, while our research star ranges from 7 to 8 (Table 3). Since these studies (28–32) were retrospective cohort studies, none of them received a star for item 4 (Demonstration That the Outcome of Interest Was Not Present at the Start of Study). In addition, only one study (31) had an adequate follow-up period of three weeks, and the remaining studies were completed between 3 and 14 days, and thus none of these studies (26, 28–30, 32) received a star for item 7 (Was Follow-Up Long Enough for Outcomes to Occur). In general, in all studies, patients can be representative of exposed populations and are from the same population as the non-exposed, with fully recorded medical records. Confounding factors are basically controlled, and all studies are complete without losing follow-up.

**TABLE 3:** The methodological quality of cohort studies and non-randomized controlled trials.

Study	1. Representativeness of the exposed cohort	2. Selection of the non-exposed cohort	3. Ascertainment of exposure	4. Demonstration that outcome of interest was not present at start of study	5. Comparability of cohorts on the basis of the design or analysis	6. Assessment of outcome	7. Was follow-up long enough for outcomes to occur	8. Adequacy of follow up of cohorts	Total
Chen et al. (30)		★	★	/	★★	★	/	★	7
Qu et al. (28)	★	★	★	/	★★	★	/	★	7
Chen et al. (32)	★	★	★	/	★★	★	/	★	7
Xia et al. (21)	★	★	★	/	★★	★	★	★	8
Qu et al. (29)	★	★	★	/	★★	★	/	★	7
Xiao et al. (26)	★	★	★	★	★★	★	/	★	8

The symbol * indicates the scores.

The IHE QA checklist was employed to evaluate the methodological quality of case series studies. One study (27) was rated 11 and the other (34) was rated 9. In general, cases in both studies were collected at a single center, and outcome assessors were not blinded. The follow-up time of a study (27) was 3–7 days, and a study (34) did not report a course of treatment, generating the problem that important events and outcomes could not be adequately observed. For the statistics of the results, the random variability of relevant outcomes was not estimated. Neither competing interests nor sources of support were reported (Supplementary Table 5).

### Certainty of evidence

The certainty of evidence for meta-analysis outcomes was assessed using the GRADE approach and categorized as “very low,” “low,” “moderate,” and “high.” Overall, most of the outcomes of meta-analysis were rated as “low” quality due to the observational study design. Among efficacy outcomes, the evidence certainty of nucleic acid negative conversion time and the cure rate were “moderate,” while the nucleic acid negative conversion rate, total effective rate, and CT improvement rate were “low.” In the outcomes of disappearance time of clinical symptoms, except for the evidence certainty of fever was “moderate” and “very low” of cough, nasal congestion and rhinorrhea, all others (including diarrhea, fatigue and pharyngalgia) were “low.” Among the evidence certainty of laboratory outcomes, CRP was “moderate,” IL6, Lym, and Neu were “low,” and LYM and WBC were “very low” (Supplementary Table 6).

### Efficacy outcomes

#### Nucleic acid negative conversion rate

**FIGURE 2:**
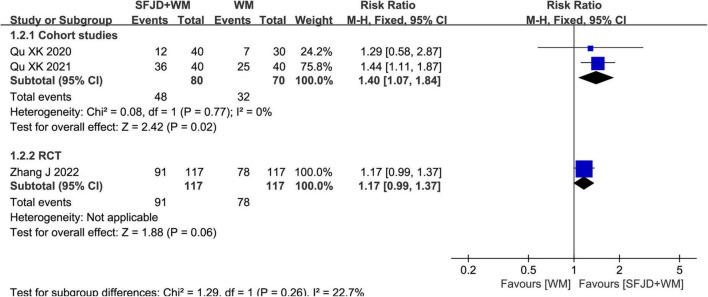
Meta-analysis of nucleic acid conversion rate in the SFJD + WM group vs. the WM group in COVID-19 patients.

Two cohort studies (28, 29) and a RCT (33) reported the rate of nucleic acid negative conversion. Based on meta-analysis results of cohort studies, SFJD + WM group had obviously elevated nucleic acid negative conversion rate compared with WM group (*RR* = 1.40, 95%CI: 1.07–1.84, *P* = 0.02). Nevertheless, the results of the RCT (*RR* = 1.17, 95%CI: 0.99 to 1.37, *P* = 0.06) were not statistically significant (Figure 2).

#### Nucleic acid negative conversion time

**FIGURE 3:**
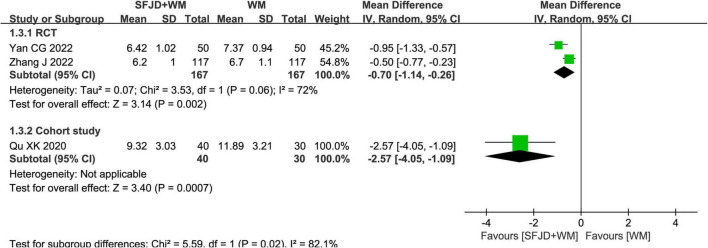
Meta-analysis of nucleic acid conversion time in the SFJD + WM group vs. the WM group in COVID-19 patients.

The nucleic acid conversion time was reported in two RCTs and one cohort study. The results of the RCTs (25, 33) demonstrated that the SFJD + WM could shorten the time of nucleic acid negative conversion, compared with the WM (*MD* = −0.70 days, 95%CI: −1.14 to −0.26, *P* = 0.002). While that of the cohort study (29) revealed that compared with the non-exposed group, the nucleic acid negative conversion time of the SFJD + WM group was shorter (*MD* = −2.57 days, 95%CI: −4.05 to −1.09, *P* = 0.0007) (Figure 3).

#### Total effective rate

**FIGURE 4:**
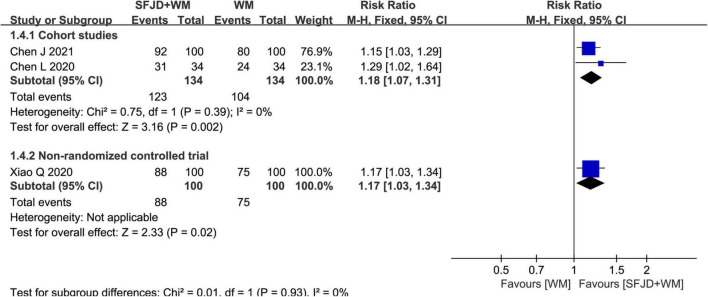
Meta-analysis of total effective rate in the SFJD + WM group vs. the WM group in COVID-19 patients.

There were 2 cohort studies (30, 32) and 1 non-RCT (26) reporting total effective rate. The results of both cohort studies (*RR* = 1.18, 95%CI: 1.07–1.31, *P* = 0.002, cohort study) and non-RCT (*RR* = 1.17, 95%CI: 1.03–1.34, *P* = 0.02, non-RCT) revealed that SFJD + WM group had markedly superior total effective rate to WM group (Figure 4).

#### The cure rate

**FIGURE 5:**
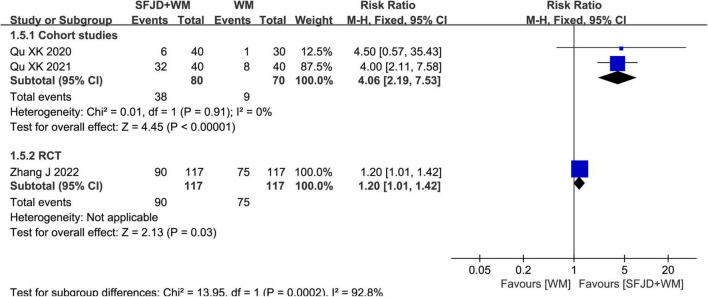
Meta-analysis regarding the cure rate between SFJD + WM and WM groups among COVID-19 cases.

Two cohort studies (28, 29) and a RCT (33) compared the cure rates. The results showed that SFJD + WM group could increase the cure rate relative to WM group (*RR* = 4.06, 95%CI: 2.19–7.53, *P* < 0.00001, cohort studies) (*RR* = 1.20, 95%CI: 1.01–1.42, *P* = 0.03, RCT) (Figure 5).

#### The rate of turning to severe/critical illness

The results of a RCT (33) and a cohort study (30) revealed that adding SFJD on the basis of WM could reduce the rate of turning to severe/critical illness, but the difference had no statistical significance between the SFJD + WM and the WM group (*RR* = 0.14, 95%CI: 0.01–2.74, *P* = 0.20, RCT) (*RR* = 0.33, 95%CI: 0.10–1.13, *P* = 0.08, cohort study) (Table 4).

**TABLE 4:** Comparison of efficacy outcomes in the SFJD + WM group vs. the WM group in COVID-19 patients.

Outcome	Type of study	Number of study	Sample size (E/C)	Statistical method	Effect estimate (95%CI)	*P*-value	Included studies
The rate of turning to severe/critical illness	RCT	1	117/117	RR	0.14 [0.01, 2.74]	0.20	Zhang et al. (33)
	Cohort study	1	34/34	RR	0.33 [0.10, 1.13]	0.08	Chen et al. (30)
The length of hospital stay	Cohort study	1	34/34	MD	–0.82 [–2.71, 1.07]	0.40	Chen et al. (30)

E, Experiment group; C, Control group; RCT, Randomized controlled trial; RR, Risk Ratio; MD, Mean Difference.

#### The chest CT improvement rate

**FIGURE 6:**
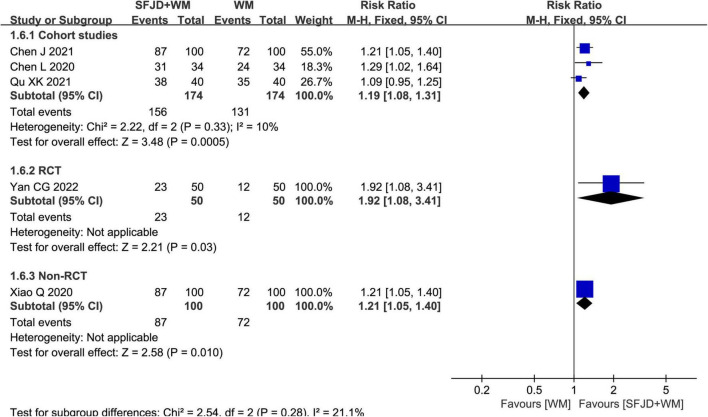
Meta-analysis regarding the CT improvement rate between SFJD + WM and WM groups among COVID-19 cases.

Meta-analysis results from three cohort studies (28, 30, 32) manifested that SFJD could significantly increase chest CT improvement rate compared with WM group (*RR* = 1.19, 95%CI: 1.08–1.31, *P* = 0.0005, cohort studies). A RCT (25) and a non-randomized controlled study (26) also found that SFJD + WM group achieved an increased chest CT improvement rate compared with control group (*RR* = 1.92, 95%CI: 1.08–3.41, *P* = 0.03, RCT) (*RR* = 1.21, 95%CI: 1.05–1.40, *P* = 0.01, non-RCT) (Figure 6).

#### The length of hospital stay

There was only one article reporting the length of hospital stay (30), and the result showed that SFJD + WM could reduce the length of hospital stay relative to the WM group while the difference was not statistically significant (*MD* = −0.82 days, 95%CI: −2.71 to 1.07, *P* = 0.40) (Table 4).

### Clinical symptom outcomes

#### Time of disappearance of clinical symptoms

**FIGURE 7:**
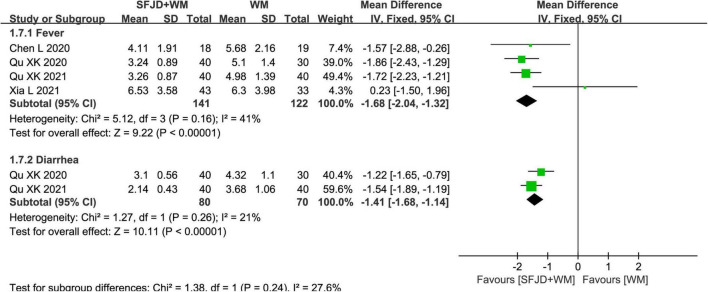
Meta-analysis on symptom disappearance time in the SFJD + WM group vs. the WM group in COVID-19 patients (fixed effect model).

**FIGURE 8:**
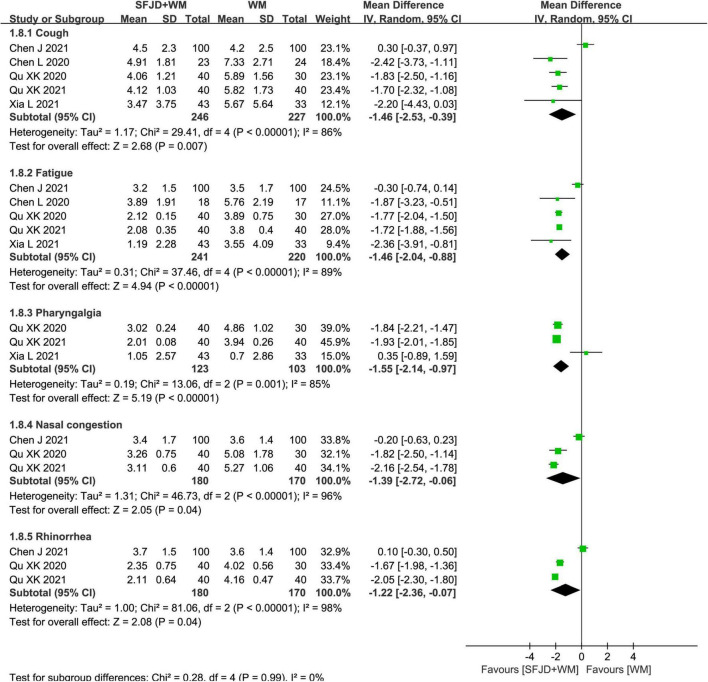
Meta-analysis on symptom disappearance time in the SFJD + WM group vs. the WM group in COVID-19 patients (random effect model).

In cohort studies, the effects of SFJD on clinical symptoms were compared. Therefore, compared with WM group, the SFJD + WM group could shorten the disappearance time of fever (*MD* = −1.68 days, 95%CI: −2.04 to −1.32, *P* < 0.00001), diarrhea (*MD* = −1.41 days, 95%CI: −1.68 to −1.14, *P* < 0.00001), cough (*MD* = −1.46 days, 95%CI: −2.53 to −0.39, *P* = 0.007), fatigue (*MD* = −1.46 days, 95%CI: −2.04 to −0.88, *P* < 0.00001), pharyngalgia (*MD* = −1.55 days, 95%CI: −2.14 to −0.97, *P* < 0.00001), nasal congestion (*MD* = −1.39 days, 95%CI: −2.72 to −0.06, *P* = 0.04), rhinorrhea (*MD* = −1.22 days, 95%CI: −2.36 to −0.07, *P* = 0.04) and expectoration (*MD* = −3.96 days, 95%CI: −5.80 to −2.12, *P* < 0.0001) (Figures 7, 8 and Supplementary Table 7). As for the time of constipation and dizziness, differences between two groups were of no significance (*P* ≥ 0.05) (Supplementary Table 7). Since the *I*^2^ of meta-analyses about cough, fatigue, nasal congestion and runny nose were all greater than 50%, we tried to explored the source of heterogeneity. When one study (Chen et al. (32) was removed, *I*^2^ could be reduced to less than 50%, which might be related to the WM intervention (only arbidol used) in this article.

The results of the RCT (25) and the non-RCT (26) revealed that the time of disappearance of fever in the SFJD + WM group decreased compared with WM group (*MD* = −0.90 days, 95%CI: −1.37 to −0.43, *P* = 0.0002, RCT) (*MD* = −0.83 days, 95%CI: −1.22 to −0.44, *P* < 0.0001, non-RCT). Differences in time of disappearance of cough, fatigue, dizziness, nasal congestion and rhinorrhea were not significant (*P* ≥ 0.05) (Supplementary Table 7).

#### Rate of disappearance of clinical symptoms

As reported in one article (30), compared with the non-exposed group, SFJD + WM could better improve the disappearance rate of cough (*RR* = 1.69 days, 95%CI: 1.14–2.49, *P* = 0.009), expectoration (*RR* = 2.41 days, 95%CI: 1.04–5.57, *P* = 0.04) and fatigue (*RR* = 1.40 days, 95%CI: 1.02–1.92, *P* = 0.04), except for fever with no statistical significance (*P* ≥ 0.05) (Supplementary Table 8).

A case series study (27) reported the disappearance rate of fever (Rate: 0.89, 95%CI: 0.73–0.97), cough (Rate: 0.80, 95%CI: 0.63–0.92) and fatigue (Rate: 0.91, 95%CI: 0.76–0.98) among COVID-19 cases receiving SFJD plus WM treatment.

### Laboratory outcomes

**FIGURE 9:**
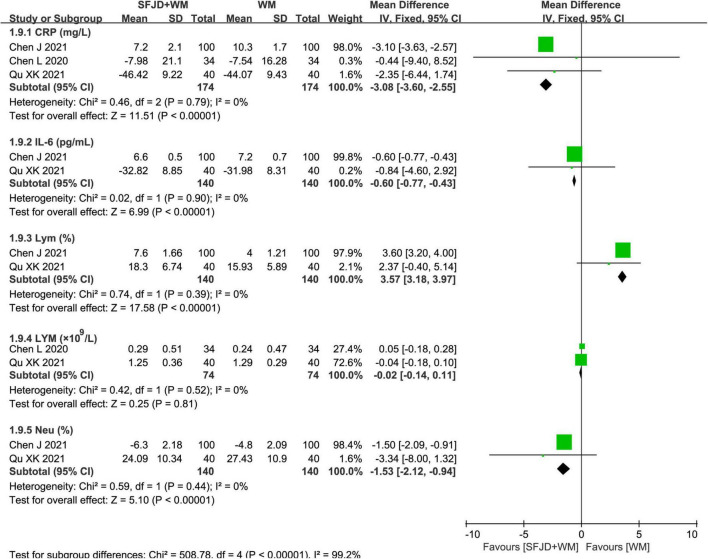
Meta-analysis on laboratory outcomes in the SFJD + WM group vs. the WM group in COVID-19 patients (fixed effect model).

**FIGURE 10:**
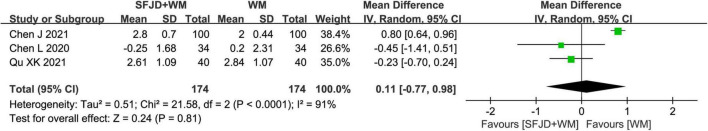
Meta-analysis on WBC in the SFJD + WM group vs. the WM group in COVID-19 patients (random effect model).

The meta-analysis results of the cohort studies showed that SFJD + WM was conducive to the normalization of CRP (*MD* = −3.08 mg/l, 95%CI: −3.60 to −2.55, *P* < 0.00001), IL-6 (*MD* = −0.60 pg/ml, 95%CI: −0.77 to −0.43, *P* < 0.00001), Lym (*MD* = 3.57%, 95%CI: 3.18 to 3.97, *P* < 0.00001) and Neu (*MD* = −1.53%, 95%CI: −2.12 to −0.94, *P* < 0.00001) (Figure 9). Additionally, there existed no significant differences in WBC, LYM, D-imer, ESR, LDH, NEUT, PA, PCT, and PLA between the SFJD + WM and WM groups (*P* ≥ 0.05) (Figure 10 and Supplementary Table 9).

From the results of both an RCT (25) and a non-RCT (26), it could be found that SFJD + WM improved WBC (*MD* = 1.42 × 10^9^/L, 95%CI: 0.93–1.91, *P* < 0.00001, RCT) (*MD* = 1.04 × 10^9^/L, 95%CI: 0.58–1.50, *P* < 0.0001, non-RCT) and Lym (*MD* = 3.54%, 95%CI: 2.61–4.47, *P* < 0.00001, RCT) (*MD* = 2.15%, 95%CI: 0.98–3.32, *P* = 0.0003, non-RCT) levels better than WM treatment (Supplementary Table 9).

### Safety outcomes

A total of nine studies reported adverse events, with one study (27) reporting no adverse events and eight studies (25, 26, 28–33) reporting adverse events including nausea, vomiting, sour regurgitation, abdominal pain, bloating, abnormal increase of liver enzymes, allergic reaction, chest tightness, loss of appetite, headache and myocardial injury. As presented in Table 5, gastrointestinal reactions were the main adverse reactions, and results demonstrated that adverse drug reactions were not significantly different between SFJD + WM and WM groups (*P* > 0.05).

**TABLE 5:** Comparison of safety outcomes in the SFJD + WM group vs. the WM group in COVID-19 patients.

Outcome	Type of study	Number of study	Sample size (E/C)	Statistical method	Effect estimate (95%CI)	*P*-value	Included studies
Nausea	Cohort study	3	114/104	RR	1.12 [0.34, 3.75]	0.86	Chen et al. (30); Qu et al. (28, 29)
	RCT	1	117/117	RR	1.40 [0.46, 4.29]	0.56	Zhang et al. (33)
Vomiting	RCT	1	117/117	RR	0.75 [0.17, 3.28]	0.70	Zhang et al. (33)
Sour regurgitation	Cohort study	1	34/34	RR	0.33 [0.01, 7.91]	0.50	Chen et al. (30)
Abdominal pain	RCT	1	117/117	RR	1.20 [0.38, 3.82]	0.76	Zhang et al. (33)
	Cohort study	1	100/100	RR	1.00 [0.14, 6.96]	1.00	Chen et al. (32)
Bloating	Cohort study	1	40/40	RR	0.33 [0.01, 7.91]	0.50	Qu et al. (28)
Abnormal increase of liver enzymes	Cohort study	1	34/34	RR	0.33 [0.01, 7.91]	0.50	Chen et al. (30)
Allergic reaction	RCT	1	50/50	RR	0.50 [0.05, 5.34]	0.57	Yan et al. (25)
	Cohort study	1	100/100	RR	0.50 [0.05, 5.43]	0.57	Chen et al. (32)
Chest tightness	Non-RCT	1	100/100	RR	0.50 [0.05, 5.43]	0.57	Xiao et al. (26)
Loss of appetite	RCT	1	117/117	RR	1.25 [0.34, 4.54]	0.73	Zhang et al. (33)
Headache	RCT	1	117/117	RR	0.50 [0.13, 1.95]	0.32	Zhang et al. (33)
Myocardial injury	RCT	1	50/50	RR	2.00 [0.19, 21.36]	0.57	Yan et al. (25)

E, Experiment group; C, Control group; RCT, randomized control trial; RR, Risk Ratio.

### Meta-regression

Meta-regression was conducted to test the reliability of the pooled analysis and search for possible causes for this heterogeneity. We found that language and male-female ratio had no statistically significant effect on the disappearance time of cough, while the sample size might affect it. Meanwhile, the results of disappearance time of fatigue were influenced by male-female ratio. Language and sample size were not the source of significant heterogeneity (Supplementary Table 10).

### Publication bias

As the number of studies in any comparative analysis did not over 10 articles, the publication bias was not evaluated.

## Discussion

### Summary of evidence

An extensive systematic review was made to evaluate the efficacy and safety of SFJD combined with WM for COVID-19. Nine databases and clinical trial registries were systemically searched. Finally, 10 clinical trials involving 1,083 cases were enrolled into this work. Our results prove that SFJD + WM exerts a significant therapeutic impact on mild and ordinary COVID-19 cases, as evidenced by the enhanced negative conversion rate of nucleic acid detection, increased cure and total effective rates, increased chest CT improvement rate, shortened nucleic acid negative conversion time, shortened clinical symptom disappearance time (fever, diarrhea, cough, fatigue, pharyngalgia, nasal congestion, and rhinorrhea), as well as improved levels of laboratory outcomes (CRP, IL-6, Lym, and Neu). Nevertheless, there was no statistical significance in turning to severe/critical illness rate, length of hospital stay, WBC, etc. Furthermore, the incidence of adverse reactions was not significant between two groups.

### Possible mechanism of Shufeng Jiedu capsule

Shufeng Jiedu capsule consists of eight herbs, namely, the root and rhizome of *Reynoutria japonica* Houtt, the dried fruit of *Forsythia suspensa* (Thunb.) Vahl, the dried root of *Isatis tinctoria* L, the dried root of *Bupleurum chinense* DC, the dried whole grass of *Patrinia scabiosifolia* Fisch. ex Trevir, the dry aboveground part of *Verbena officinalis* L, the fresh or dried rhizome of *Phragmites communis* Trin, the dried root and rhizome of *Glycyrrhiza uralensis* Fisch (35). The results of an article integrating absorption, distribution, metabolism and excretion (ADME) evaluation, network establishment, target estimation as well as functional bioinformatics analysis also demonstrate that SFJD can modulate immunomodulation as well as anti-inflammation associated targets involved in several pathways by the effective ingredients, suggesting the possible efficacy against novel coronavirus (36). According to fundamental research, SFJD decreases viral load within bilateral lungs in HCOV-229E mice, and decreases inflammatory factors in the lungs (31). Additionally, it has antiviral, anti-inflammatory, antipyretic, and immunomodulatory effects. A study (37) initially explored the overall effect of SFJD through microarray, finding that G protein-coupled receptor 18 (GPR18) might be involved in the signaling pathway. They further identified the chemical components of SFJD, which found that verbenalin was a potential anti-inflammatory active ingredient, as well as confirmed its anti-inflammatory effect via GPR18 in GPR18 knockout mice.

### Comparison with other studies or reviews

A review (38) analyzed whether SFJD was effective and safe in treating acute exacerbations of chronic obstructive pulmonary disease (COPD). Based on their observations, SFJD was possibly more beneficial for decreasing the treatment failure rate, and shortening hospital stays while improving symptoms. According to the findings of researcher (39), SFJD might reduce clinical symptom duration, improve cure rates in patients with acute upper respiratory tract infections, and be safe to the administer. Currently, there are only three registered clinical protocols for systemically investigating the effect of SFJD on treating COVID-19, while the results have not been published. Therefore, our study is the first to comprehensively report the efficacy of SFJD capsule in treating COVID-19.

### Implication for practice

Most of the COVID-19 patients included in our study were classified as mild and ordinary types. SFJD is a recommended drug during the “medical observation period” according to “Diagnosis and Treatment Protocol for Novel Coronavirus Pneumonia.” Furthermore, our study summarized the therapeutic effect of SFJD on patients diagnosed with mild and ordinary types of COVID-19.

There are many research reports regarding the application of TCM in the treatment of COVID-19 (40). “3 medicines” (Jinhua Qinggan granule, LianhuaQingwen capsule/granule, Xuebijing) and “3 formulations” (Qingfei Paidu decoction, Huashi Baidu formula and XuanFei Baidu granule) are effective representative drugs in the treatment of COVID-19 in China. Results of a systematic review of “3 medicines” and “3 formulations” treatment for COVID-19 demonstrated some statistically significant effects on symptoms, chest CT manifestations, laboratory outcomes and length of stay (41). Our study exhibited similar therapeutic effects, which might expand the scope of clinical applications of SFJD and provide a treatment plan for the treatment of COVID-19.

### Strengths and limitations

This is the first study that assesses the efficacy and safety of SFJD combined with WM for treating COVID-19. Several different types of study designs were included, hoping to provide more integrative data supporting the application of SFJD in treating COVID-19. Nine databases and clinical trial registries were searched, and more detailed and accurate retrieval strategies were adopted for minimizing the potential publication bias. In addition, the COS of COVID-19 clinical studies, incorporating outcomes of patient concerns was used, which might provide more valuable references for the clinical application of SFJD.

Certain limitations should be noted in this work. Firstly, most studies have methodological flaws in design that may affect our results. Secondly, only Chinese and English literatures were included, which might cause a potential language bias. Thirdly, results of protocols that have been registered with clinical trial registries have not been published, which may be limited from published status. Finally, the low sample size made it impossible to conduct subgroup analysis on the severity of COVID-19 patients and the use of different WM groups.

## Conclusion

To conclude, this study provides evidence that the combination of SFJD with WM can bring benefit to COVID-19 patients, including improve the outcomes of efficacy, laboratory and clinical symptoms. Nonetheless, more well-designed RCTs are still warranted.

## Data availability statement

The original contributions presented in this study are included in the article/Supplementary material, further inquiries can be directed to the corresponding author.

## Author contributions

XS, JHZ, and LG were involved in the conceptualization. XS and CC wrote the original draft. JHZ and LG reviewed and edited the article. XS, CC, and DZ completed the literature retrieval. HH, GJ, ZG, and HW performed the data extraction. XS, ZJ, and JBZ analyzed the data and evaluated the risk of bias. All authors have read, contributed, and approved the manuscript.

## Abbreviations

ADME: absorption distribution metabolism excretion
COVID-19: coronavirus disease 2019
COS: core outcome sets
CKNI: China National Knowledge Infrastructure
CI: confidence interval
COPD: chronic obstructive pulmonary disease
CT: computerized tomography
IHE: Institute of Health Economics
MD: mean difference
NOS: Newcastle-Ottawa Scale
PRISMA: Preferred Reporting Items for Systematic Reviews and Meta-Analyses
QA: quality appraisal
RR: Risk ratio
ROB: Risk of Bias
RCT: Randomized controlled trial
SARS-CoV-2: severe acute respiratory syndrome coronavirus-2
SFJD: Shufeng Jiedu Capsule
TCM: Traditional Chinese medicine
WM: Western medicine
WHO: The World Health Organization.
