# Supplementary material for: The add-on effect of Shufeng Jiedu capsule for treating COVID-19: A systematic review and meta-analysis
Source: Front Med (Lausanne). 2022 Oct 13;9:1020286. doi: 10.3389/fmed.2022.1020286 (PMC9620801; doi:10.3389/fmed.2022.1020286)
Supplement: Supplementary file 1 [file Table_1.DOCX]

**Table S1. Search strategy**

| **Step** | **Searchs** | **Number** |
| --- | --- | --- |
| **Pubmed** | | |
| #1 | "COVID-19"[Mesh] OR "SARS-CoV-2"[Mesh] OR "SARS-CoV-2 variants" [Supplementary Concept] | 177747 |
| #2 | COVID-19[Title/Abstract] OR COVID 19[Title/Abstract] OR 2019-nCov[Title/Abstract] OR SARS-CoV-2[Title/Abstract] OR 2019 novel coronavirus[Title/Abstract] OR coronavirus disease 2019[Title/Abstract] OR coronavirus disease-19[Title/Abstract] OR "severe acute respiratory syndrome coronavirus 2"[Title/Abstract] OR "new coronavirus"[Title/Abstract] | 267528 |
| #3 | #1 OR #2 | 276712 |
| #4 | "Shufeng Jiedu"[Title/Abstract] OR "ShufengJiedu"[Title/Abstract] OR "shufengjiedu"[Title/Abstract] | 48 |
| #5 | #3 AND #4 | 27 |
| **Cochrane Library** | | |
| #1 | MeSH descriptor: [COVID-19] explode all trees | 2116 |
| #2 | ('novel coronavirus pneumonia'):ti,ab,kw OR ('COVID-19'):ti,ab,kw OR ('COVID-2019'):ti,ab,kw OR ('SARS-CoV-2'):ti,ab,kw OR ('coronavirus disease-19'):ti,ab,kw OR ('COVID19'):ti,ab,kw OR ('2019 novel coronavirus infection'):ti,ab,kw OR ('coronavirus disease 2019'):ti,ab,kw OR ('2019 nCoV disease'):ti,ab,kw OR ('2019 novel coronavirus disease'):ti,ab,kw OR ('2019 nCoV infection'):ti,ab,kw OR ('Wuhan coronavirus'):ti,ab,kw OR ('Wuhan seafood market pneumonia virus'):ti,ab,kw OR ('coronavirus disease 2019 virus'):ti,ab,kw OR ('SARS2'):ti,ab,kw | 11696 |
| #3 | ('Shufeng Jiedu'):ti,ab,kw OR ('ShufengJiedu'):ti,ab,kw OR ('shufengjiedu'):ti,ab,kw | 17 |
| #4 | #1 OR #2 | 11696 |
| #5 | #3 AND #4 | 3 |
| **Web of science** | | |
| #1 | TS= (COVID-19 OR COVID 19 OR 2019-nCov OR SARS-CoV-2 OR 2019 novel coronavirus OR coronavirus disease 2019 OR coronavirus disease-19 OR severe acute respiratory syndrome coronavirus 2 OR new coronavirus OR coronavirus disease 2019) AND TS= (Shufeng Jiedu OR ShufengJiedu OR shufengjiedu) | 30 |
| **Embase** | | |
| #1 | 'coronavirus disease 2019'/exp OR 'severe acute respiratory syndrome coronavirus 2'/exp | 265506 |
| #2 | 'COVID-19':ab,ti OR 'COVID 19':ab,ti OR '2019-nCov':ab,ti OR 'SARS-CoV-2':ab,ti OR '2019 novel coronavirus':ab,ti OR 'coronavirus disease 2019':ab,ti OR 'coronavirus disease-19':ab,ti OR 'severe acute respiratory syndrome coronavirus 2':ab,ti OR 'new coronavirus':ab,ti OR 'coronavirus disease 2019':ab,ti | 283411 |
| #3 | #1 OR #2 | 311239 |
| #4 | 'Shufeng Jiedu':ab,ti OR 'ShufengJiedu':ab,ti OR 'shufengjiedu':ab,ti | 70 |
| #5 | #3 AND #4 | 30 |
| **WHO COVID-19 database** | | |
| #1 | Title, abstract, subject: Shufeng Jiedu OR ShufengJiedu OR shufengjiedu | 30 |
| **China National Knowledge Infrastructure (CKNI)** | | |
| #1 | (SU=2019冠状病毒 OR SU=新型冠状病毒 OR SU=新冠肺炎 OR SU=冠状病毒肺炎 OR SU=新冠疫情 OR SU=新冠病毒 OR SU=2019-nCoV肺炎 OR SU=2019-nCoV OR SU=SARS-CoV-2 OR SU=Novel coronavirus OR SU=nCoV OR SU=Emerging Coronaviruses OR SU=new coronavirus OR SU=COVID-19 OR SU=coronavirus) AND (SU=疏风解毒胶囊) | 41 |
| **Wanfang Data Knowledge Service platform** | | |
| #1 | 主题:(2019冠状病毒 OR 新型冠状病毒 OR 新冠肺炎 OR 冠状病毒肺炎 OR 新冠疫情 OR 新冠病毒 OR 2019-nCoV肺炎 OR 2019-nCoV OR SARS-CoV-2 OR Novel coronavirus OR nCoV OR Emerging Coronaviruses OR new coronavirus OR COVID-19 OR coronavirus) AND 主题:(疏风解毒胶囊) | 35 |
| **VIP information resource integration service platform** | | |
| #1 | (M=2019冠状病毒 OR M=新型冠状病毒 OR M=新冠肺炎 OR M=冠状病毒肺炎 OR M=新冠疫情 OR M=新冠病毒 OR M=2019-nCoV肺炎 OR M=2019-nCoV OR M=SARS-CoV-2 OR M=Novel coronavirus OR M=nCoV OR M=Emerging Coronaviruses OR M=new coronavirus OR M=COVID-19 OR M=coronavirus) AND (M=疏风解毒胶囊) | 21 |
| **Chinese Biological Medicine database (CBM)** | | |
| #1 | ("2019冠状病毒"[常用字段:智能] OR "新型冠状病毒"[常用字段:智能] OR "新冠肺炎"[常用字段:智能] OR "冠状病毒肺炎"[常用字段:智能] OR "新冠病毒"[常用字段:智能] OR "2019-nCoV肺炎"[常用字段:智能] OR "新冠疫情"[常用字段:智能] OR "2019-nCoV"[常用字段:智能] OR "SARS-CoV-2"[常用字段:智能] OR "Novel coronavirus"[常用字段:智能] OR "nCoV"[常用字段:智能] OR "Emerging Coronaviruses"[常用字段:智能] OR "new coronavirus"[常用字段:智能] OR "COVID-19"[常用字段:智能] OR "coronavirus"[常用字段:智能]) AND ("疏风解毒胶囊"[不加权:扩展] OR "疏风解毒胶囊"[常用字段:智能]) | 44 |
