# Supplementary material for: The add-on effect of Shufeng Jiedu capsule for treating COVID-19: A systematic review and meta-analysis
Source: Front Med (Lausanne). 2022 Oct 13;9:1020286. doi: 10.3389/fmed.2022.1020286 (PMC9620801; doi:10.3389/fmed.2022.1020286)
Supplement: Supplementary file 2 [file Table_2.DOCX]

**Table S2. The reasons and lists of studies excluded in full-text screen**

| **Study** | **Reason for Exclude** |
| --- | --- |
| Wang Z 2020 (1) | case report |
| Zhuang W 2020 (2) | multi traditional Chinese medicine |
| Wu Y 2021 (3) | multi traditional Chinese medicine |
| Wang W 2021 (4) | multi traditional Chinese medicine |
| Cao R 2020 (5) | multi traditional Chinese medicine |
| Lyu M 2021 (6) | multi traditional Chinese medicine |
| Zhang T 2020 (7) | experimental study |
| Lu H 2020 (8) | experimental study |
| Zhang TJ 2020 (9) | experimental study |
| Li C 2020 (10) | experimental study |
| Chen L 2020 (11) | not traditional Chinese medicine |
| Tang F 2020 (12) | not traditional Chinese medicine |

**REFERENCES**

1. Wang Z, Chen X, Lu Y, Chen F, Zhang W. Clinical characteristics and therapeutic procedure for four cases with 2019 novel coronavirus pneumonia receiving combined Chinese and Western medicine treatment. *Biosci Trends*. (2020) 14: 64-8. doi: 10.5582/bst.2020.01030

2. Zhuang W, Fan Z, Chu Y, Wang H, Yang Y, Wu L, et al. Chinese patent medicines in the treatment of coronavirus disease 2019 (COVID-19) in china. *Front Pharmacol*. (2020) 11. doi: 10.3389/fphar.2020.01066

3. Wu Y, Zhong P. Clinical progress on management of pneumonia due to COVID-19 with chinese traditional patent medicines. *Front Pharmacol*. (2021) 12. doi: 10.3389/fphar.2021.655063

4. Wang W, Xie Y, Zhou H, Liu L. Contribution of traditional Chinese medicine to the treatment of COVID-19. *Phytomedicine*. (2021) 85. doi: 10.1016/j.phymed.2020.153279

5. Cao R, Ma Q, Xu Q, Duan H, Du Lidong, Ning Y, et al. Potential common mechanism of four chinese patent medicines recommended by diagnosis and treatment protocol for COVID-19 (Trial version 7) in medical observation period. *Pharmacology and Clinics of Chinese Materia Medica*. (2020) 36: 2-8. doi: 10.13412/j.cnki.zyyl.20200603.006

6. Lyu M, Fan G, Xiao G, Wang T, Xu D, Gao J, et al. Traditional Chinese medicine in COVID-19. *Acta Pharm Sin B*. (2021) 11: 3337-63. doi: 10.1016/j.apsb.2021.09.008

7. Zhang T, Xu J, Shen X, Han Y, Liu J, Zhang H, et al. Basic study on treatment of COVID-19 with Shufeng Jiedu Capsule and research and development ideas of new Chinese materia medica against COVID-19. *Chinese Traditional and Herbal Drugs*. (2020) 51: 2273-82. doi: 10.7501/j.issn.0253-2670.2020.09.001

8. Lu H. Drug treatment options for the 2019-new coronavirus (2019-nCoV). *Biosci Trends*. (2020) 14: 69-71. doi: 10.5582/bst.2020.01020

9. Zhang TJ, Xu J, Shen XP, Han YQ. Basic study on treatment of COVID-19 with Shufeng Jiedu Capsule and research and development ideas of new Chinese materia medica against COVID-19. *Chinese Traditional and Herbal Drugs*. (2020) 51: 2273-82. doi: 10.7501/j.issn.0253-2670.2020.09.001

10. Li C, Wang L, Ren L. Antiviral mechanisms of candidate chemical medicines and traditional Chinese medicines for SARS-CoV-2 infection. *Virus Res*. (2020) 286. doi: 10.1016/j.virusres.2020.198073

11. Chen L, Cheng ZQ, Liu F, Xia Y, Chen YG. [Analysis of 131 cases of COVID-19 treated with Ganlu Xiaodu Decoction]. *Zhongguo Zhong Yao Za Zhi*. (2020) 45: 2232-8. doi: 10.19540/j.cnki.cjcmm.20200322.505

12. Tang F, Fan XY. Clinical analysis of 364 outpatients with fever during epidemic period of COVID-19. *Jiangsu Medical Journal*. (2020) 46: 541-5. doi: 10.19460/j.cnki.0253-3685.2020.06.001
