# Supplementary material for: The add-on effect of Shufeng Jiedu capsule for treating COVID-19: A systematic review and meta-analysis
Source: Front Med (Lausanne). 2022 Oct 13;9:1020286. doi: 10.3389/fmed.2022.1020286 (PMC9620801; doi:10.3389/fmed.2022.1020286)
Supplement: Supplementary file 4 [file Table_4.DOCX]

**Table S4. Diagnostic criteria of included study**

| **Study** | **Diagnostic criteria** |
| --- | --- |
| Xia L 2021 (1) | Diagnosis and Treatment Protocol for Coronavirus Pneumonia (NA) |
| Chen J 2021 (2) | Diagnosis and Treatment Protocol for Coronavirus Pneumonia (Trial version 6) |
| Chen L 2020 (3) | Diagnosis and Treatment Protocol for Coronavirus Pneumonia (Trial version 7) |
| Qu XK 2021 (4) | Diagnosis and Treatment Protocol for Coronavirus Pneumonia (Trial version 6) |
| Qu XK 2020 (5) | Diagnosis and Treatment Protocol for Coronavirus Pneumonia (Trial version 5) |
| Wu Y 2021 (6) | Diagnosis and Treatment Protocol for Coronavirus Pneumonia (Trial version 6) |
| Guo GH 2021 (7) | Diagnosis and Treatment Protocol for Coronavirus Pneumonia (Trial version 5) |
| Xiao Q 2020 (8) | Diagnosis and Treatment Protocol for Coronavirus Pneumonia (Trial version 5) |
| Yan CG 2022 (9) | Diagnosis and Treatment Protocol for Coronavirus Pneumonia (NA) |
| Zhang J 2022 (10) | Diagnosis and Treatment Protocol for Coronavirus Pneumonia (NA) |

NA= no apply

**REFERENCES**

1. Xia L, Shi Y, Su J, Friedemann T, Tao Z, Lu Y, et al. Shufeng Jiedu, a promising herbal therapy for moderate COVID-19:Antiviral and anti-inflammatory properties, pathways of bioactive compounds, and a clinical real-world pragmatic study. *Phytomedicine*. (2021) 85: 153390. doi: 10.1016/j.phymed.2020.153390

2. Chen J, Lin S, Niu C, Xiao Q. Clinical evaluation of Shufeng Jiedu Capsules combined with umifenovir (Arbidol) in the treatment of common-type COVID-19: A retrospective study. *Expert Rev Resp Med*. (2021) 15: 257-65. doi: 10.1080/17476348.2020.1822741

3. CHEN L, LIU F, WU JH, SONG HY, XIA JS, SHENG B, et al. Clinical efficacy of shufeng jiedu capsule combined with western medicine in treatment of common COVID-19 patients by retrospective analysis. *Chinese Journal of Experimental Traditional Medical Formulae*. (2020) 26: 14-20. doi: 10.13422/j.cnki.syfjx.20201628

4. QU XK, TANG C, HAO SL, MA JH, WEI GY, SONG KY, et al. Observation on the clinical effect of Shufeng Jiedu capsule combined with Arbidol in the treatment of NPC. *Journal of China Prescription Drug*. (2021) 19: 6-8. doi: 10.3969/j.issn.1671-945X.2021.03.004

5. QU XK, HAO SL, MA JH, WEI GY, SONG KY, TANG C, et al. Observation on clinical effect of Shufeng Jiedu Capsule combined with Arbidol Hydrochloride Capsule in treatment of COVID-19. *Chinese Traditional and Herbal Drugs*. (2020) 51: 1167-70. doi: 10.7501/j.issn.0253-2670.2020.05.011

6. WU Y, XIAO Y, GONG ZH. A retrospective analysis of shufeng jiedu capsules combined therapy in treating corona virus disease 2019. *World Latest Medicine Information (Electronic Version)*. (2021): 40-1, 48. doi: 10.3969/j.issn.1671-3141.2021.18.014

7. GUO GH, SONG B, ZHU CQ, ZHANG QH, YE KL, XIAO JH. Retrospective clinical analysis of traditional chinese medicine shufeng jiedu capsule combined with montelukast in the treatment of corona virus disease 2019. *World Latest Medicine Information (Electronic Version)*. (2021) 21: 19-23. doi: 10.3969/j.issn.1671-3141.2021.10.006

8. XIAO Q, JIANG YJ, WU SS, WANG Y, AN J, XU WP, et al. Value analysis of Shufeng Jiedu Capsule combined with Arbidol in the treatment of mild COVID-19. *Journal of emergency in Chinese traditional Medicine*. (2020) 29: 756-8. doi: 10.3969/j.issn.1004-745X.2020.05.002

9. YAN CG, SHAN HX, PEI XG, LU RJ, WU YZ, WEN Q. Efficacy and safety analysis of shufeng jiedu capsules combined with interferon alpha plus arbidol in the treatment of common COVID-19. *Journal of Guangzhou University of Traditional Chinese Medicine*. (2022) 39: 475-80. doi: 10. 13359/j. cnki. gzxbtcm. 2022. 03. 003

10. Zhang J, Liu L, Zhang G, Li M, Ma B, Yang W. Treating patients infected with the SARS-CoV-2 Omicron variant with a traditional Chinese medicine, Shufeng Jiedu capsule. *Biosci Trends*. (2022) 16: 238-41. doi: 10.5582/bst.2022.01220
