# Supplementary material for: The add-on effect of Shufeng Jiedu capsule for treating COVID-19: A systematic review and meta-analysis
Source: Front Med (Lausanne). 2022 Oct 13;9:1020286. doi: 10.3389/fmed.2022.1020286 (PMC9620801; doi:10.3389/fmed.2022.1020286)
Supplement: Supplementary file 5 [file Table_5.DOCX]

| **Table S5.** The methodological quality of case-series studies was evaluated by IHE QA checklist | | | |
| --- | --- | --- | --- |
| **Study** | | Wu Y 2021 (1) | Guo GH 2021 (2) |
| **Criterion** | | Rating | |
|  |  | Yes; Partial/unclear; No | |
| Study objective | | | |
| 1 | Was the hypothesis/aim/objective of the study clearly stated? | Y | Y |
| Study design | |  |  |
| 2 | Was the study conducted prospectively? | Y | N |
| 3 | Were the cases collected in more than one center? | N | N |
| 4 | Were patients recruited consecutively? | N | N |
| Study population | | |  |
| 5 | Were the characteristics of the patients included in the study described? | Y | Y |
| 6 | Were the eligibility criteria (i.e. inclusion and exclusion criteria) for entry into the study clearly stated? | Y | N |
| 7 | Did patients enter the study at a similar point in the disease? | Y | Y |
| Intervention and cointervention | | |  |
| 8 | Was the intervention of interest clearly described? | Y | N |
| 9 | Were additional interventions (cointerventions) clearly described? | Y | N |
| Outcome measures | | |  |
| 10 | Were relevant outcome measures established a priori? | Y | Y |
| 11 | Were outcome assessors blinded to the intervention that patients received? | N | N |
| 12 | Were the relevant outcomes measured using appropriate objective/subjective methods? | N | Y |
| 13 | Were the relevant outcome measures made before and after the intervention? | N | Y |
| Statistical analysis | | |  |
| 14 | Were the statistical tests used to assess the relevant outcomes appropriate? | N | Y |
| Results and conclusions | | |  |
| 15 | Was follow-up long enough for important events and outcomes to occur? | N | N |
| 16 | Were losses to follow-up reported? | Y | Y |
| 17 | Did the study provided estimates of random variability in the data analysis of relevant outcomes? | N | N |
| 18 | Were the adverse events reported? | Y | N |
| 19 | Were the conclusions of the study supported by the results? | Y | Y |
| Competing interests and sources of support | | |  |
| 20 | Were both competing interests and sources of support for the study reported? | N | N |
| **Total** | | 11 | 9 |

**REFERENCES**

1. WU Y, XIAO Y, GONG ZH. A retrospective analysis of shufeng jiedu capsules combined therapy in treating corona virus disease 2019. *World Latest Medicine Information (Electronic Version)*. (2021): 40-1, 48. doi: 10.3969/j.issn.1671-3141.2021.18.014

2. GUO GH, SONG B, ZHU CQ, ZHANG QH, YE KL, XIAO JH. Retrospective clinical analysis of traditional chinese medicine shufeng jiedu capsule combined with montelukast in the treatment of corona virus disease 2019. *World Latest Medicine Information (Electronic Version)*. (2021) 21: 19-23. doi: 10.3969/j.issn.1671-3141.2021.10.006
