# Supplementary material for: The add-on effect of Shufeng Jiedu capsule for treating COVID-19: A systematic review and meta-analysis
Source: Front Med (Lausanne). 2022 Oct 13;9:1020286. doi: 10.3389/fmed.2022.1020286 (PMC9620801; doi:10.3389/fmed.2022.1020286)
Supplement: Supplementary file 6 [file Table_6.DOCX]

**Table S6.** GRADE Certainty of the Evidence Summary Table

| Outcomes | No. of studies | Study design | No. of Participants | | Effect | | Certainty of the Evidence |
| --- | --- | --- | --- | --- | --- | --- | --- |
|  |  |  | SFJD+WM | WM | Relative  (95% CI) | Absolute  (95% CI) |  |
| Nucleic acid negative conversion rate | 2 | Cohort study | 48/80 (60.0%) | 32/70 (45.7%) | RR 1.40  (1.07 to 1.84) | 183 more per 1,000  (from 32 more to 384 more) | Low |
| Nucleic acid negative conversion time | 2 | RCT | 167 | 167 | / | MD 0.7 lower  (1.14 lower to 0.26 lower) | Moderate |
| Total effective rate | 2 | Cohort study | 123/134 (91.8%) | 104/134 (77.6%) | RR 1.18  (1.07 to 1.31) | 140 more per 1,000  (from 54 more to 241 more | Low |
| The cure rate | 2 | Cohort study | 38/80 (47.5%) | 9/70 (12.9%) | RR 4.06  (2.19 to 7.53) | 393 more per 1,000  (from 153 more to 840 more) | Moderate |
| The CT improvement rate | 3 | Cohort study | 156/174 (89.7%) | 131/174 (75.3%) | RR 1.19  (1.08 to 1.31) | 143 more per 1,000  (from 60 more to 233 more) | Low |
| The disappearance time of fever | 4 | Cohort study | 141 | 122 | / | MD 1.68 lower  (2.04 lower to 1.32 lower) | Moderate |
| The disappearance time of diarrhea | 2 | Cohort study | 80 | 70 | / | MD 1.41 lower  (1.68 lower to 1.14 lower) | Low |
| The disappearance time of cough | 5 | Cohort study | 246 | 227 | / | MD 1.46 lower  (2.53 lower to 0.39 lower) | Very low |
| The disappearance time of fatigue | 5 | Cohort study | 241 | 220 | / | MD 1.46 lower  (2.04 lower to 0.88 lower) | Low |
| The disappearance time of pharyngalgia | 3 | Cohort study | 123 | 103 | / | MD 1.55 lower  (2.14 lower to 0.97 lower) | Low |
| The disappearance time of nasal congestion | 3 | Cohort study | 180 | 170 | / | MD 1.39 lower  (2.72 lower to 0.06 lower) | Very low |
| The disappearance time of rhinorrhea | 3 | Cohort study | 180 | 170 | / | MD 1.22 lower  (2.36 lower to 0.07 lower) | Very low |
| CRP (mg/L) | 3 | Cohort study | 174 | 174 | / | MD 3.08 lower  (3.6 lower to 2.55 lower) | Moderate |
| IL-6 (pg/mL) | 2 | Cohort study | 140 | 140 | / | MD 0.6 lower  (0.77 lower to 0.43 lower) | Low |
| Lym (%) | 2 | Cohort study | 140 | 140 | / | MD 3.57 higher  (3.18 higher to 3.97 higher) | Low |
| LYM (×10^9^/L) | 2 | Cohort study | 74 | 74 | / | MD 0.02 lower  (0.14 lower to 0.11 higher) | Very low |
| Neu (%) | 2 | Cohort study | 140 | 140 | / | MD 1.53 lower  (2.12 lower to 0.94 lower) | Low |
| WBC (×10^9^/L) | 3 | Cohort study | 174 | 174 | / | MD 0.11 higher  (0.77 lower to 0.98 higher) | Very low |

RCT, Randomized controlled trial; RR: Risk Ratio; MD: Mean Difference; WM: Western medicine; SFJD: Shufeng Jiedu Capsule
