# Supplementary material for: The add-on effect of Shufeng Jiedu capsule for treating COVID-19: A systematic review and meta-analysis
Source: Front Med (Lausanne). 2022 Oct 13;9:1020286. doi: 10.3389/fmed.2022.1020286 (PMC9620801; doi:10.3389/fmed.2022.1020286)
Supplement: Supplementary file 7 [file Table_7.DOCX]

**Table S7.** Comparison of symptom disappearance time in the SFJD + WM group *vs*. the WM group in COVID-19 patients

| Outcome | Type of  study | Number of  study | Sample Size(E/C) | Statistical method | Effect estimate (days) (95%CI) | P-  value | Included  studies |
| --- | --- | --- | --- | --- | --- | --- | --- |
| Fever disappearance time | RCT | 1 | 50/50 | MD | -0.90[-1.37, -0.43] | 0.0002 | Yan CG 2022 |
|  | Non-RCT | 1 | 100/100 | MD | -0.83[-1.22, -0.44] | <0.0001 | Xiao Q 2020 |
| Cough disappearance time | RCT | 2 | 167/167 | MD | -0.53[-1.77, 0.72] | 0.41 | Yan CG 2022  Zhang J 2022 |
|  | Non-RCT | 1 | 100/100 | MD | 0.28[-0.40, 0.96] | 0.42 | Xiao Q 2020 |
| Fatigue disappearance time | RCT | 2 | 167/167 | MD | -0.80[-1.63, 0.02] | 0.06 | Yan CG 2022  Zhang J 2022 |
|  | Non-RCT | 1 | 100/100 | MD | -0.33[-0.78, 0.12] | 0.15 | Xiao Q 2020 |
| Dizziness disappearance time | RCT | 1 | 50/50 | MD | -0.23[-0.83, 0.37] | 0.46 | Yan CG 2022 |
|  | Non-RCT | 1 | 100/100 | MD | 0.18[-0.31, 0.67] | 0.48 | Xiao Q 2020 |
| Nasal congestion disappearance time | RCT | 1 | 50/50 | MD | -0.27[-0.76, 0.22] | 0.28 | Yan CG 2022 |
|  | Non-RCT | 1 | 100/100 | MD | -0.17[-0.61, 0.27] | 0.45 | Xiao Q 2020 |
| Rhinorrhea disappearance time | RCT | 1 | 50/50 | MD | -0.15[-0.74, 0.44] | 0.62 | Yan CG 2022 |
|  | Non-RCT | 1 | 100/100 | MD | 0.08[-0.33, 0.49] | 0.70 | Xiao Q 2020 |
| Constipation disappearance time | Cohort study | 1 | 100/100 | MD | -0.10[-0.31, 0.11] | 0.35 | Chen J 2021 |
| Expectoration disappearance time | Cohort study | 1 | 34/34 | MD | -3.96[-5.80, -2.12] | <0.0001 | Chen L 2020 |
| Dizziness disappearance time | Cohort study | 1 | 100/100 | MD | 0.20[-0.27, 0.67] | 0.41 | Chen J 2021 |

E: Experiment group; C: Control group; RCT: Randomized controlled trial; MD: Mean Difference
