# Supplementary material for: The add-on effect of Shufeng Jiedu capsule for treating COVID-19: A systematic review and meta-analysis
Source: Front Med (Lausanne). 2022 Oct 13;9:1020286. doi: 10.3389/fmed.2022.1020286 (PMC9620801; doi:10.3389/fmed.2022.1020286)
Supplement: Supplementary file 8 [file Table_8.DOCX]

**Table S8.** Comparison of symptom disappearance rate in COVID-19 patients

| Outcome | Type of study | Number of study | Sample Size  (E/C) | Statistical  method | Effect estimate  (95%CI) | P-value | Included  studies |
| --- | --- | --- | --- | --- | --- | --- | --- |
| Fever disappearance rate | Cohort study | 1 | 34/34 | RR | 1.26[0.98, 1.61] | 0.07 | Chen L 2020 |
| Cough disappearance rate | Cohort study | 1 | 34/34 | RR | 1.69[1.14, 2.49] | 0.009 | Chen L 2020 |
| Expectoration disappearance rate | Cohort study | 1 | 34/34 | RR | 2.41[1.04, 5.57] | 0.04 | Chen L 2020 |
| Fatigue disappearance rate | Cohort study | 1 | 34/34 | RR | 1.40[1.02, 1.92] | 0.04 | Chen L 2020 |

E: Experiment group; C: Control group; RR: Risk Ratio
