# Supplementary material for: The add-on effect of Shufeng Jiedu capsule for treating COVID-19: A systematic review and meta-analysis
Source: Front Med (Lausanne). 2022 Oct 13;9:1020286. doi: 10.3389/fmed.2022.1020286 (PMC9620801; doi:10.3389/fmed.2022.1020286)
Supplement: Supplementary file 9 [file Table_9.DOCX]

**Table S9.** Comparison of laboratory outcomes in the SFJD + WM group *vs*. the WM group in COVID-19 patients

| Outcome | Type of study | Number of  study | Sample Size  (E/C) | Statistical  method | Effect estimate  (95%CI) | P-value | Included  studies |  |  |
| --- | --- | --- | --- | --- | --- | --- | --- | --- | --- |
| WBC(×10^9^/L) | RCT | 1 | 50/50 | MD | 1.42[0.93, 1.91] | <0.00001 | Yan CG 2022 |  |  |
|  | Non-RCT | 1 | 100/100 | MD | 1.04[0.58, 1.50] | <0.0001 | Xiao Q 2020 |  |  |
| Lym (%) | RCT | 1 | 50/50 | MD | 3.54[2.61, 4.47] | <0.00001 | Yan CG 2022 |  |  |
|  | Non-RCT | 1 | 100/100 | MD | 2.15[0.98, 3.32] | 0.0003 | Xiao Q 2020 |  |  |
| D-imer (mg/L) | Cohort study | 1 | 34/34 | MD | -0.37[-0.96, 0.22] | 0.22 | Chen L 2020 |  |  |
| ESR (mm/h) | Cohort study | 1 | 40/40 | MD | 2.63[-0.80, 6.06] | 0.13 | Qu XK 2021 |  |  |
| LDH (U/L) | Cohort study | 1 | 40/40 | MD | 14.62[-8.06, 37.30] | 0.21 | Qu XK 2021 |  |  |
| NEU (×10^9^/L) | Cohort study | 1 | 34/34 | MD | -0.37[-1.30, 0.56] | 0.44 | Chen L 2020 |  |  |
| PA (mg/L) | Cohort study | 1 | 40/40 | MD | 7.98[-6.75, 22.71] | 0.29 | Qu XK 2021 |  |  |
| PCT ($\boldsymbol{\mu}$g/L) | Cohort study | 1 | 34/34 | MD | 0.21[-0.18, 0.60] | 0.30 | Chen L 2020 |  |  |
| PLA (×10^9^/L) | Cohort study | 1 | 100/100 | MD | 3.40[-9.50, 16.30] | 0.61 | Chen J 2021 |  |  |

E: Experiment group; C: Control group; RCT: Randomized controlled trial; MD: Mean Difference
