# Supplementary material for: The add-on effect of Shufeng Jiedu capsule for treating COVID-19: A systematic review and meta-analysis
Source: Front Med (Lausanne). 2022 Oct 13;9:1020286. doi: 10.3389/fmed.2022.1020286 (PMC9620801; doi:10.3389/fmed.2022.1020286)
Supplement: Supplementary file 10 [file Table_10.DOCX]

| **The disappearance time of cough** | | | | | | |
| --- | --- | --- | --- | --- | --- | --- |
| **logRR** | **Coef.** | **Std. Err.** | **z** | **p** | **[95% Conf. Interval]** | |
| Intrcpt | -3.5860 | 0.9569 | -3.7476 | 0.0002 | -5.4615 | -1.7106 |
| Language | -0.5850 | 1.2134 | -0.4822 | 0.6297 | -2.9633 | 1.7932 |
| Sample size | 0.0201 | 0.0094 | 2.1456 | 0.0319 | 0.0017 | 0.0385 |
| Male-female ratio | 0.2435 | 0.6746 | 0.3609 | 0.7181 | -1.0787 | 1.5658 |
| **The disappearance time of** **fatigue** | | | | | | |
| **logRR** | **Coef.** | **Std. Err.** | **z** | **p** | **[95% Conf. Interval]** | |
| Intrcpt | -2.9663 | 1.0665 | -2.7813 | 0.0054 | -5.0566 | -0.8760 |
| Language | 0.0023 | 0.0277 | 0.0812 | 0.9353 | -0.0521 | 0.0566 |
| Sample size | -0.4361 | 0.7738 | -0.5636 | 0.5731 | -1.9526 | 1.0805 |
| Male-female ratio | 0.0148 | 0.0066 | 2.2524 | 0.0243 | 0.0019 | 0.0276 |

**Table S10**.The Meta-regression for the disappearance time of cough and fatigue
